# Study protocol for a randomized controlled trial to evaluate a web-based comprehensive sexual health and media literacy education program for high school students

**DOI:** 10.1186/s13063-019-3992-1

**Published:** 2020-01-08

**Authors:** Tracy M. Scull, Christina V. Malik, Abigail Morrison, Elyse M. Keefe

**Affiliations:** Innovation Research & Training, 5316 Highgate Drive, Suite 121, Durham, NC 27713 USA

**Keywords:** Randomized controlled trial, Media literacy education, Adolescents, Sexual health education, Web-based program

## Abstract

**Background:**

School-based comprehensive sexual health education can improve adolescent health outcomes, and web-based programs are a promising approach to overcoming challenges associated with teacher-led formats by ensuring that students receive content that is consistent, unbiased, and medically accurate. However, many adolescents do not receive high-quality sexual health education and turn to the media for information about sex and relationships. Consumption of sexual media messages is related to early and risky sexual behaviors. Media literacy education (MLE) is a proven approach to adolescent sexual health promotion, yet there are no rigorously evaluated web-based MLE programs to promote sexual and relationship health among high school students.

**Methods:**

This study will test the efficacy, in a randomized controlled trial, of *Media Aware*, a web-based comprehensive sexual health promotion program for high school students that uses an MLE approach. Participants will be students in 9th and 10th grade health classes in participating schools. Randomization will take place at the school level, and data collection will take place at three time points (i.e., pretest, posttest, and 3 months follow-up). Students in the intervention classrooms will receive *Media Aware* between pretest and posttest, and students in the delayed-intervention classrooms will receive *Media Aware* after study completion (i.e., after 3 months follow-up data collection). Students in the delayed-intervention classes will receive their standard health education programming, and teachers in the delayed-intervention classes will be asked to refrain from teaching sexual health or MLE during the study timeframe. The primary outcome variables are intentions, willingness, and behaviors related to sexual health and sexual activity.

**Discussion:**

There are currently no evidence-based comprehensive sexual health programs for high school students that are web-based and use an MLE approach. *Media Aware* has the potential to be an engaging, less expensive, and effective sexual and relationship health program for high school students. *Media Aware* is unique in two important ways: (1) the web-based format reduces many of the challenges to fidelity of implementation associated with teacher-led sexual health education; and (2) the MLE approach addresses a commonly ignored influence on adolescent sexual and relationship health, namely, media.

**Trial registration:**

ClinicalTrials.gov, NCT04035694. Registered on 29 July 2019.

**Contact for Scientific Queries:** Tracy Scull, PhD (Principal Investigator); innovation Research & Training at 5316 Highgate Drive, Suite 121, Durham, North Carolina, USA 27713; tscull@irtinc.us.

## Background

School-based comprehensive sexual health education programs can successfully promote adolescent sexual health [1, 2]. Evidence-based programs have been shown to delay sexual debut, reduce the number of lifetime sexual partners, increase condom use during sexual activity, and reduce sexually transmitted infections (STIs), teen pregnancy, and risky sexual behaviors [1–3]. Despite these positive outcomes, sexual health programming in the USA remains inconsistent. Less than half of states require sexual health education to be taught in schools, and just 13 states require that the information taught be medically accurate [4, 5]. Many schools are not required to implement evidence-based programs, and less than half of school districts provide professional development funding for teachers to support learning how to teach sexual health education [6], which can result in inconsistent, inaccurate, and biased instruction.

The sexual health programming that is taught in secondary education seldom includes information beyond abstinence, contraception, and HIV/STIs [5]. Topics such as healthy relationships, consent, dating violence, and gender role stereotypes all play a role in shaping adolescent sexual health and should be included in comprehensive sexual education programming [7–17]. Adolescents who do not learn about healthy relationships or consent may be at increased risk for dating violence, sexual assault, and sexual abuse [18, 19]; gender role expectations can influence youth decision-making to engage in risky sexual behavior and put gender minority youth at a greater risk for poor health outcomes [9, 20]. Therefore, there is a need for comprehensive evidence-based programs for sexual and relationship health promotion among adolescents that can be implemented with fidelity in schools.

An important influence on adolescent sexual health that is often overlooked in sexual health education is media influence. Teens spend an average of 9 h a day using entertainment media [21]. They are exposed to copious amounts of sexual content, which is often inaccurate, incomplete, and unhealthy [22]. Media have been found to influence adolescent sexual attitudes, perceptions, and behaviors [23], and can even act as a “super peer” during sexual identity development [24, 25]. Exposure to sexually explicit material is linked to stricter gender role attitudes, more permissive sexual norms [26], increased support for the objectification of women, increased acceptance of violence against women, rape myths, and sexual harassment [23]. Youth exposed to sexual media are also more likely to report having had sex than youth who have little to no exposure [27, 28].

Media literacy education (MLE) has been shown to be an effective pedagogical approach to sexual and relationship health promotion. MLE aims to improve critical thinking skills so that individuals do not simply accept media messages but, instead, evaluate them for realism and accuracy. MLE has been shown to positively affect youth cognitions including their media knowledge, criticism, and attitudes [29, 30]. Youth who receive MLE are more aware that media perpetuate inaccurate sexual messages than youth who do not receive MLE [31, 32]. MLE has been shown to redress teens’ inaccurate normative beliefs about their peers, including norms about the frequency of sexual activity and abstinence [31, 33, 34]. In studies assessing MLE’s effect on health behaviors, youth receiving MLE reported higher levels of self-efficacy regarding their own sexual behavior [31, 35], increased intentions to use a condom, if they decide to have sex [32], and reductions in risky sexual behaviors [34].

Given barriers to implementing traditional sexual health education programs and the commonly narrow focus of curricula that exclude information about the important influence of media on sexual health, there is a need for a comprehensive sexual health education program that integrates education about media influence and does not rely on teacher-led instruction for delivery. In order to address the many important topics frequently omitted from traditional sexual health education programs, including media influence, and to reduce variability in the content presented across instructors, *Media Aware*, a web-based media literacy and comprehensive sexual health education program, was developed. The program is designed to teach media literacy skills, reduce risky sexual behavior among high school students, and positively affect attitudes and cognitions that promote sexual health. *Media Aware* is designed to be completed individually by students using a web-based application that is self-paced, thereby allowing students to control the pace of their own learning. The web-based nature of the program also means that the educational content is standardized for all learners and free from individual teacher bias. The main objective of this study is to evaluate both the immediate and short-term effectiveness of *Media Aware* on adolescent sexual health and media-related outcomes. The study will also help to evaluate factors associated with fidelity of implementation and adoption, including acceptance and satisfaction as reported by both teachers and students.

## Methods/design

### Aim, design, and setting

The study design is a two-arm, randomized controlled trial (intervention group and delayed-intervention group) that will be used to evaluate the effectiveness of *Media Aware*, a web-based MLE and sexual health education program designed for high school students. It is expected that approximately 16 schools, with one teacher and their 9th or 10th grade classes from each school, will participate in the study. Data will be collected from students at three time points (i.e., pretest, immediate posttest, and 3 months follow-up). Primary outcomes will include students’ sexual behaviors, intentions, and willingness to engage in sexual activities. Secondary outcomes will include attitudes and cognitions related to sexual health (e.g., rape myth acceptance). Additionally, program completion and feedback on satisfaction using the program will be collected from both teachers and intervention students. It is expected that up to 1600 students may participate in the study. This study will take place during the 2019–2020 school year. The Standard Protocol Items: Recommendations for Interventional Trials (SPIRIT) checklist is provided as Additional file 1.

#### Sample size and power calculation

Based upon a desired power of .80, a small-to-medium effect size (.40, as defined by Cohen [36]), cluster size (participating students per school) = 100, and an intraclass correlation coefficient (ICC) for school effects = .05, a power analysis (α = .05) revealed that about 15 schools are needed to participate [37]. Attrition between pretest-posttest in a small feasibility study conducted previously was very low (< 4%); this study aimed to recruit an additional school (*N* = 16) to participate to address possible attrition. The power analysis will be updated as schools are recruited to determine if any additional schools need to be recruited to achieve the desired power.

#### Internal Review Board approval

This study protocol (Version 1) was approved by the innovation Research & Training Internal Review Board (IRB) chaired by Dr. Barbara Davis Goldman on April 29, 2019. The protocol was assigned IRB number 19-002-1-EFF.

### Participant characteristics

Recruitment will take place at three levels: schools, teachers, and students. Teacher participants will be health teachers from each of the participating schools. One teacher per school will be eligible to participate. Teachers must lead at least one 9th or 10th grade health class. *Media Aware* will be evaluated with 9th and 10th grade students, who are provided with parent permission and who also provide their assent, from the classes of each of the participating teachers.

### Processes

#### Schools

High schools from across the USA will be recruited for participation. If required, school district permission for conducting external research projects in the school will be obtained prior to participation. School district administrators will receive access to *Media Aware* and study questionnaires to review, if requested. Schools will be recruited through health teachers, school district administrators, or prevention specialists. Recruitment methods of communication will include health education listservs, emails, Facebook or other Internet ads, flyers at conference booths or presentations, and phone calls or word of mouth. Efforts will be made to balance inclusion of both public and private schools and urban and rural schools, and schools ranging in their socioeconomic composition. Based on the initial power analysis, approximately 16 schools are expected to participate. Schools will be eligible if they have at least one 9th or 10th grade health classroom.

#### Teachers

One teacher from each of the participating schools will be recruited for study participation. Teachers may be recruited after a school has indicated interest in the study or may be recruited directly without prior contact with the school’s administrators. Teachers will participate with up to five of their health classrooms consisting of 9th or 10th grade students. It is anticipated that if class sizes average 20 students, up to 1600 students may be recruited. Teachers will be eligible if they can teach health education to 9th or 10th grade students, can provide technology (e.g., computers or other Wi-Fi-enabled devices) to their students for use during class to complete the web-based questionnaires and course, and are proficient in English. Teachers will review and complete an informed consent form prior to participation in the study. Participating teachers will need to agree to be randomly assigned to either the intervention or the delayed-intervention group; both groups involve implementing *Media Aware* in their classrooms. Because of the potentially sensitive nature of the program content (i.e., sexual health education), teachers will need to have access to the program during the recruitment process to review the content in order to make a decision about using the program with their classes.

#### Students

Schools will implement their district policies regarding student participation in sexual health education (e.g., opt in, opt out). Parents may request to review *Media Aware* as part of the decision-making process.

It is expected that up to 1600 students in the 9th or 10th grades may be recruited to participate in this study. Project staff and/or participating teachers will introduce the study to students and distribute consent forms. Students will be eligible to participate if they are members of participating teachers’ classes, are enrolled in 9th or 10th grade, are fluent in English, and have appropriate parental permission to receive sexual health education (per district policies). Parents can receive access to study questionnaires to review, if requested, prior to making the decision to allow their child’s participation in the research study. Students and their parents will complete assent and permission forms, respectively, and affirmative parent permission and student assent must be received before study participation begins (IRB-approved assent and permission forms are available on ClinicalTrials.gov). Consent forms specify that student participant data will be accessible to project staff only, used anonymously, and will not be sold to any outside party. The forms also state that students can stop participation at any time. This study does not involve collecting biological specimens for storage.

Informed consent will be obtained in accordance with 21 Code of Federal Regulations (CFR) Part 50 and the Declaration of Helsinki before protocol-specified procedures are carried out. The protocol is also designed to meet the Health and Human Services Regulations regarding the Protection of Human Subjects 45 CFR 46 – Subpart D.

#### Protocol

Any changes to the study protocol will be submitted by the Principal Investigator (PI) to the IRB for approval and subsequently updated in the clinical trial registry. Changes that affect the study design, budget, or timeline will be made in conjunction with the PI, the research team, and the funder. After IRB approval, the PI will distribute a copy of the revised protocol to the research team (e.g., data collectors) and ensure that all research team members are aware of the revisions that have been made. Any unexpected deviations from the protocol will be documented by the research team. With respect to the Data and Safety Monitoring Plan (DSMP), the National Institutes of Health (NIH) Program Officer will be notified of any updates, and changes will not take effect without NIH approval.

#### The intervention

**Fig. 1:**
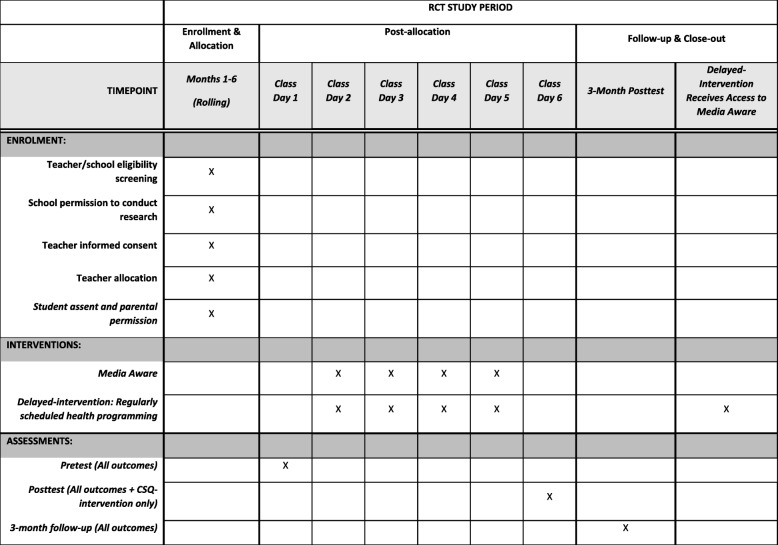
SPIRIT figure of enrollment, interventions, and assessments

*Media Aware* is a web-based, comprehensive, sexual health education program for high school students that provides developmentally appropriate and medically accurate sexual health information using an MLE approach. The pairing of sexual health information and MLE allows the program to teach students how to think critically about media messages. It especially teaches them what information is frequently omitted from media messages, such as the potential consequences of sexual activity, consent, and examples of healthy relationships. *Media Aware* consists of four interactive lessons, each containing two or three smaller mini-lessons. Each overarching lesson is designed to be completed in a 45-min class period; thus, the program is designed to take a total of four standard class periods to complete. *Media Aware* was developed by a multidisciplinary team consisting of professionals in the fields of developmental psychology, media research, web applications development, multimedia design, instructional design, and adolescent sexual health. The students in the intervention group will receive access to *Media Aware* in the time period between completing the pretest and the posttest (see Fig. 1).

*Media Aware* contains a Teacher Dashboard designed for use by classroom teachers, so that they are able to enroll their students in the program, track student progress, send electronic permission forms (for sexual health instruction *not* research forms), and enroll parents in a demo of the program. Teachers will also have access to a web-based Teacher Training program to help them prepare to facilitate inclusive, trauma-informed, sexual health education discussions. All teachers will receive access to *Media Aware*, the Teacher Dashboard, and the Teacher Training program. Teachers will be asked to review all of the instructional materials and associated resources.

#### Delayed-intervention group

During the course of the study, students in the delayed-intervention group will receive regularly scheduled health programming, but teachers will be asked to refrain from teaching any sexual health or MLE topics. While teachers in the delayed-intervention group may receive access to *Media Aware*, the Teacher Dashboard, and the Teacher Training prior to the student pretest, they will be instructed not to provide the program to their students until after study completion (i.e., collection of student data at 3 months follow-up).

#### Randomization

Randomization will take place at the school level to avoid contamination effects. Each school and its participating classrooms will be assigned to either the intervention or the delayed-intervention group using an open label randomized controlled trial study design. Randomization may be stratified to balance groups between public or private schools, urban or rural schools, and the average socioeconomic status level of students in schools. The research team will use Excel’s random number generator function to randomize the schools to conditions. Randomization will take place after teachers provide informed consent and appropriate school approvals for conducting the study are obtained.

#### Implementation procedure

Participating teachers will receive access to the web-based Teacher Dashboard, which will give them access to *Media Aware* and a Teacher Training program. Teachers will use the dashboard to enroll participating students in *Media Aware* and keep track of their progress. They will have the option to use the other features of the dashboard (e.g., electronic parent permission to receive sexual health education), if they choose.

This study will collect data from participating students in both study arms at three time points: pretest, posttest, and at a 3 months follow-up. All classes will be visited by project staff to administer the pretest. To ensure confidentiality, students will be spread out around the classroom and will be given privacy shields to block their computer or tablet screens from view. Secret ID numbers will be used in place of names on the questionnaires and will be sealed in envelopes between data collection time points. Students will be instructed that they can skip any questions they would like and that they may withdraw from the study at any time for any reason. Pretest questionnaires will be completed using computers or other Wi-Fi-enabled devices. Paper copies of the pretest questionnaire will be provided upon request or if technical problems with the online questionnaire occur. After pretest data collection is completed, teachers in the intervention group will have students who have permission to receive sexual health education complete *Media Aware* over the course of about one week (depending upon the school’s class schedule). It is anticipated that the program could be completed in four 45-min class periods. Teachers in the delayed-intervention schools will be asked to refrain from teaching sexual health or MLE until after the 3 months follow-up data collection. Project staff will return to all classrooms to administer the posttest questionnaire to students in the intervention group after completion of the *Media Aware* program (approximately four class periods), and to students in the delayed-intervention group approximately four class periods after their pretests have been completed. At posttest, participating students in the intervention group will also provide program feedback. After program implementation, teachers in the intervention group will provide feedback on their experiences. Project staff will return to the classrooms after approximately 3 months to administer the follow-up questionnaire. After the follow-up data are collected, teachers in the delayed-intervention group can use *Media Aware* with their students. Afterward, teachers in the delayed-intervention group will also be asked to provide feedback on their experiences implementing *Media Aware* with their students. There will be no special criteria for discontinuing or modifying allocated interventions. There is no anticipated harm.

Schools will receive a $1000 incentive for participating in the study. Teachers may receive up to $250 as an incentive for participating in the study including: a $30 incentive for each class that returns more than 80% of the parent permission forms (regardless of the parent’s participation decision) and a $100 incentive for reviewing the program, Teacher Dashboard, and Teacher Training, and completing the questionnaire on their own time. Students will be given small incentives at pretest, posttest, and follow-up (i.e., earbuds, sticky notes, water bottle) worth less than $10 in total. At follow-up, students who have participated in all three data collection time points will be entered into a classroom drawing for a $50 gift card. Students will have access to *Media Aware* and teachers will have access to the Teacher Dashboard and Teacher Training until the conclusion of the 2020 spring semester.

### Measures

#### Student measures

Students will complete questionnaires at three time points: pretest, posttest, and 3 months follow-up (see Fig. 1). The student questionnaire, consisting of approximately 180 items, will contain a compilation of measures containing assessment of variables that have previously been shown to be related to sexual health outcomes (e.g., attitudes, normative beliefs, behavioral intentions, willingness), as well as self-reports of sexual behaviors. In addition, the student questionnaire will include items designed to assess students’ media-related cognitions and media literacy skills, which are related to enhanced critical processing of media messages. Demographic questions will include the student’s age, grade level, recent grade point average (GPA), socioeconomic status (i.e., whether the student qualifies for free or reduced school lunch), ethnicity, race, sex, gender, parental education level, amount of media exposure, sexual content exposure in media, and involvement in a romantic relationship.

##### Student primary outcome measures

The primary outcome measures for students are the following:

1. *Sexual debut (8 items)*. Participants will be asked to indicate (1 = yes; 2 = no; 3 = unsure) their sexual experience including oral, vaginal, and anal sex (e.g., Have you ever had oral sex?). We will examine change in sexual activity between time points.
2. *Contraception and protection use (2 items)*. Skip logic will be used. Participants who indicate sexual experience will be asked on a 3-point scale (1 = yes; 2 = no; 3 = unsure) about their contraception/protection use the last time they engaged in sexual activity (e.g., Did you use a condom the last time you had sex [anal or vaginal]?).
3. *Willingness to engage in unprotected sex (1 item)*. Participants will be asked to indicate on a 4-point Likert scale (1 = very unwilling; 2 = unwilling; 3 = willing; 4 = very willing) how willing they are to engage in unprotected sex (i.e., Imagine you were with a boyfriend/girlfriend. They want to have sex, but neither of you have any form of protection. In this situation, how willing would you be to go ahead and have sex anyway?). This measure was adapted from Gibbons et al. [38].
4. *Willingness to have sex (1 item)*. Participants will be asked to indicate on a 4-point Likert scale (1 = very unwilling; 2 = unwilling; 3 = willing; 4 = very willing) how willing they are to have sex in a relationship context. (i.e., Imagine you were with a boyfriend/girlfriend and they say they love you. They want to have sex. In this situation, how willing would you be to have sex?). This measure was adapted from Gibbons et al. [38].
5. *Willingness to “hook up” against own wishes (1 item)*. Participants will be asked to indicate on a 4-point Likert scale (1 = very unwilling; 2 = unwilling; 3 = willing; 4 = very willing) how willing they are to “hook up” even if they are not sure that they really want to (i.e., Imagine you were with a boyfriend/girlfriend. They want to hook up, but you are not sure that you want to. In this situation, how willing would you be to go ahead and hook up anyway?). This measure was adapted from Scull et al. [34] and Gibbons et al. [38].
6. *Intentions to engage in sexual activity with another person (4 items)*. Participants will be asked to indicate on a 4-point Likert scale (1 = not at all likely; 2 = unlikely; 3 = likely; 4 = extremely likely) how likely they are to engage in sexual activity in the next year (e.g., How likely is it that you will have any type of sexual contact with another person [oral sex, anal sex, vaginal sex, or genital-to-genital contact] in the next year?). This measure was adapted from L’Engle et al. [39], α = .77.
7. *Intentions to use contraception/protection (3 items)*. Participants will be asked to indicate on a 4-point Likert scale (1 = not at all likely; 2 = unlikely; 3 = likely; 4 = extremely likely) how likely they are to use contraception (e.g., If you were to have vaginal or anal sex, how likely would you be to use a condom?). This measure was adapted from Jemmott and Jemmott [40].

##### Student secondary outcome measures

**Fig. 2:**
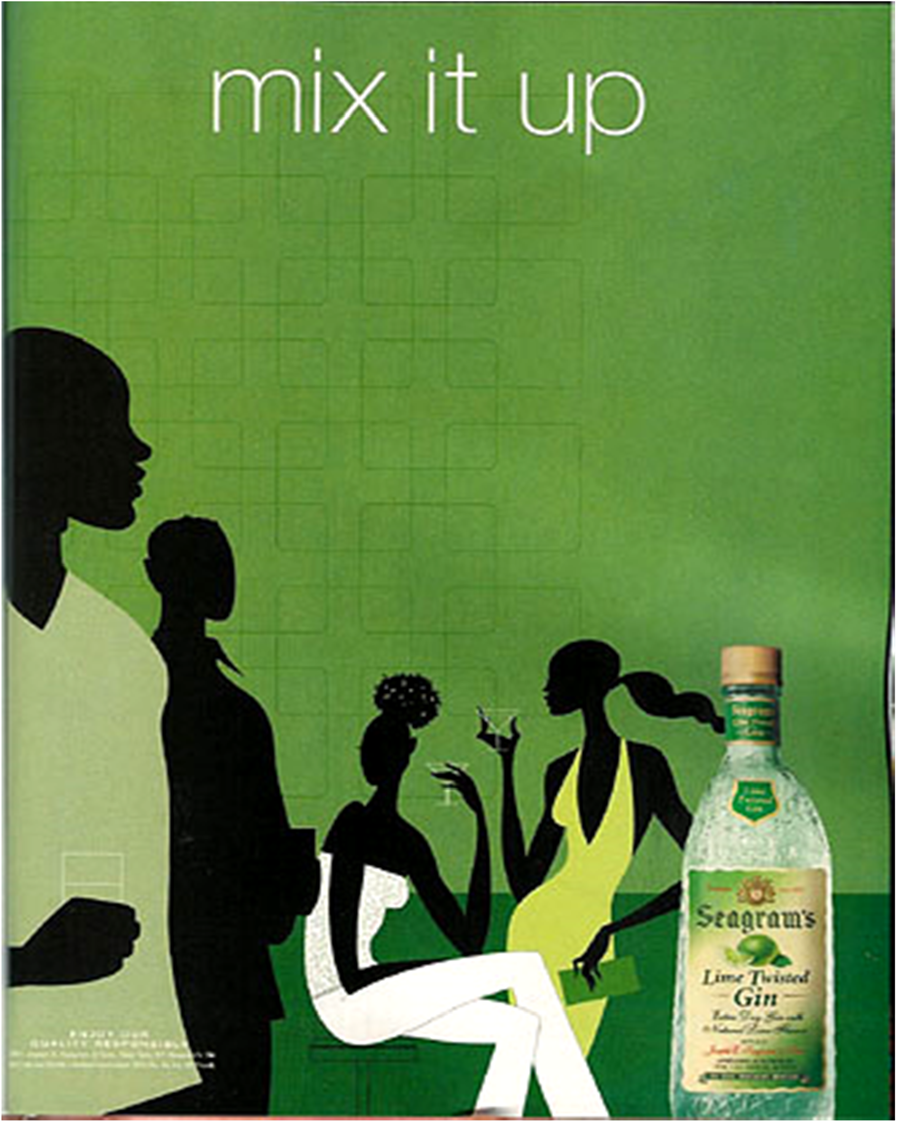
Seagram’s Lime Twisted Gin advertisement

The student secondary outcome measures are the following:

1. *Media message deconstruction skills (3 items)*. Participants will be shown an advertisement (Fig. 2) and asked to describe it in detail (e.g., How are advertisers trying to get someone to buy this product?). This measure was adapted from Kupersmidt et al. [41].
2. *Message completeness (1 item)*. Participants will be shown the same advertisement and asked to indicate on a 4-point Likert scale (1 = incomplete to 4 = complete) how complete the information included in an advertisement is (i.e., How complete is the information in this advertisement?). This measure was adapted from Scull et al. [42].
3. *Perceived realism of media messages (5 items)*. Participants will be asked to indicate on a 4-point Likert scale (1 = strongly disagree; 2 = disagree; 3 = agree; 4 = strongly agree) the degree to which they agree that media portray reality (e.g., Do teens in the media do things that average teens do?). This measure was adapted from Austin and Johnson [43], α = .75.
4. *Perceived similarity to media messages (3 items)*. Participants will be asked to indicate on a 4-point Likert scale (1 = strongly disagree; 2 = disagree; 3 = agree; 4 = strongly agree) the degree to which they agree that media portrayals are similar to the participant’s own life (e.g., The things I do in my life are similar to what I see teens do in the media.). This measure was adapted from Austin and Johnson [43], α = .79.
5. *Media skepticism (5 items)*. Participants will be asked to indicate on a 4-point Likert scale (1 = strongly disagree; 2 = disagree; 3 = agree; 4 = strongly agree) their level of agreement on several items pertaining to the media (e.g., Media are dishonest about what happens if people have sex.). This measure was adapted from Scull et al. [34], α = .86.
6. *Acceptance of dating violence (4 items)*. Participants will be asked to indicate on a 4-point Likert scale (1 = strongly disagree; 2 = disagree; 3 = agree; 4 = strongly agree) their level of agreement on several items pertaining to dating violence (e.g., It is okay for people to hit their girlfriends/boyfriends if they did something to make them mad.). This measure was adapted from Foshee et al. [44], α = .78.
7. *Acceptance of strict gender role stereotypes (6 items)*. Participants will be asked to indicate on a 4-point Likert scale (1 = strongly disagree; 2 = disagree; 3 = agree; 4 = strongly agree) their level of agreement on several items pertaining to strict gender role stereotypes (e.g., Raising children is primarily a woman’s responsibility.). This measure was adapted from Foshee et al. [44], α = .69.
8. *Acceptance of rape myths (5 items)*. Participants will be asked to indicate on a 4-point Likert scale (1 = strongly disagree; 2 = disagree; 3 = agree; 4 = strongly agree) their level of agreement on several items pertaining to myths about rape (e.g., It shouldn’t be considered rape if a guy is drunk and didn’t realize what he was doing.). This measure was adapted from Payne et al. [45] and McMahon and Farmer [46]; the overall scale reliability was .87 and the subscales ranged from .64 to .80.
9. *Efficacy to intervene as bystander (5 items)*. Participants will be asked to indicate on a scale of 0 (can’t do) to 100 (very certain) their level of confidence in several behaviors (e.g., I could talk to a friend who I suspected is in an abusive relationship.). This measure was adapted from Banyard et al. [47], α =87.
10. *Intentions to intervene as bystander (4 items)*. Participants will be asked to indicate on a 4-point Likert scale (1 = not likely at all; 2 = unlikely; 3 = likely; 4 = extremely likely) how likely they would be to intervene as a bystander to prevent sexual or relationship violence (e.g., I would approach a friend if I thought they were in an abusive relationship and let them know that I am here to help.). This measure was adapted from Banyard et al. [47], α =94.
11. *Sexual health knowledge (8 items)*. Participants will be asked several multiple choice and True/False questions about sexual health (e.g., True or False: Can you tell someone has an STI by looking at him/her? Answer: False). Some questions have more than one correct answer (e.g., Which of the following are methods of reducing the risk of pregnancy? Choose all that apply. Answer: male latex condom, birth control pill, birth control patch). Scores are a summation of correct answers; a higher score indicates a higher level of sexual health knowledge. Scores are calculated out of a possible 13 points. This measure was adapted from Scull et al. [34].
12. *Descriptive norms: teen sexual activity (5 items)*. Participants will be asked to indicate what percentage (0% = no teens; 100% = all teens) of teens engage in sexual activity (e.g., oral sex, vaginal sex, anal sex). This measure was adapted from Scull et al. [34].
13. *Descriptive norms: teen risky sexual activity (3 items)*. Participants will be asked to indicate what percentage (0% = no teens; 100% = all teens) of teens engage in risky sexual activities (e.g., What percentage of teens have sex with someone who is much older?). This measure was adapted from Scull et al. [34].
14. *Descriptive norms: birth control (3 items)*. Participants will be asked to indicate what percentage (0% = no teens; 100% = all teens) of teens use contraception (e.g., What percentage of teens who have oral sex use a condom or a dental dam?). This measure was adapted from Scull et al. [34].
15. *Descriptive norms: communication (3 items)*. Participants will be asked to indicate what percentage (0% = no teens; 100% = all teens) of teens communicate with parents, health providers, or another trusted adult (e.g., Before deciding to have sex, what percentage of teens talk with their boyfriend/girlfriend about sexual health?). This measure was adapted from Scull et al. [34].
16. *Efficacy to use contraception/protection (4 items)*. Participants will be asked to indicate on a 4-point Likert scale (1 = strongly disagree; 2 = disagree; 3 = agree; 4 = strongly agree) how effectively they could get and use contraception (e.g., If I wanted to, I could get condoms or another form of contraception.). This measure was adapted from Soet et al. [48].
17. *Efficacy to negotiate contraception/protection use (2 items)*. Participants will be asked to indicate on a 4-point Likert scale (1 = strongly disagree; 2 = disagree; 3 = agree; 4 = strongly agree) how effectively they could convince a partner to use contraception (e.g., If I decided to have sex, I could talk to any potential partner to make him/her understand why we should use condoms or other contraception.). This measure was adapted from Soet et al. [48].
18. *Efficacy to communicate before sex (3 items)*. Participants will be asked to indicate on a 4-point Likert scale (1 = strongly disagree; 2 = disagree; 3 = agree; 4 = strongly agree) how effectively they could communicate with others about sexual health (e.g., I could talk with a boyfriend/girlfriend about using condoms for sexually transmitted infection protection.). This measure was adapted from Scull et al. [32].
19. *Intent to communicate before sex (3 items)*. Participants will be asked to indicate on a 4-point Likert scale (1 = not at all likely; 2 = unlikely; 3 = likely; 4 = extremely likely) how likely they would be to communicate with others before sexual activity (e.g., Before deciding to have sex, how likely would you be to talk with your parents or another trusted adult about sexual health?). This measure was adapted from Scull et al. [32].
20. *Communication frequency (3 items)*. Participants are asked on a 4-point Likert scale (1 = never; 2 = rarely; 3 = sometimes; 4 = often) about their frequency of sexual health communication with partners or trusted adults (e.g., How often do you talk with a doctor or other health professional?).

Potential mediators and moderatorsThe following indicators are used as potential mediators:

1. *Cognitive elaboration (3 items)*. Participants will be shown the same advertisement as previously mentioned (Fig. 2) and asked to indicate on a 4-point Likert scale (1 = not much at all; 2 = a little; 3 = a good amount; 4 = a lot) the extent to which they (1) thought about the ad; (2) spent time thinking about the ad; and (3) paid attention to the advertisement (e.g., How much did you think about this advertisement?). This measure was adapted from Shiv et al. [49].
2. *Counterarguing (4 items)*. Participants will be shown the same advertisement and asked to indicate on a 4-point Likert scale (1 = not much at all; 2 = a little; 3 = a good amount; 4 = a lot) the extent to which they (1) wanted to argue back to what it was saying; (2) thought of ways in which they disagreed with what was presented; (3) thought of ways that the information being presented was inaccurate or misleading; and (4) found themselves looking for flaws in the way information was presented in the advertisement (e.g., While viewing the ad, I sometimes felt like I wanted to “argue back” to what it was saying.). This measure was adapted from Moyer-Gusé and Nabi [50], α = 84.
3. *Advertisement credibility (3 items)*. Participants will be shown an advertisement and asked to indicate on a varying 4-point Likert scale (e.g., 1 = unbelievable; 4 = believable) how (1) believable; (2) truthful; and (3) trustworthy they find the advertisement (e.g., This advertisement is unbelievable/believable.). Each Likert scale follows the above pattern. This measure was adapted from MacKenzie et al. [51].
4. *Risk prototype: hook-up against will (14 items)*. Participants will be asked to indicate which of 14 adjectives describe a person who hooks up even if they were not sure that they wanted to (e.g., Imagine someone your age who hooks up even if they are not sure that they want to. How would you describe this person, using the following characteristics: prepared, confident, desperate, etc.?). This measure was adapted from Myklestad and Rise [52] α = .89.
5. *Risk prototype: sex (14 items)*. Participants will be asked to indicate which of the same 14 adjectives describe a person who has sex with a boyfriend/girlfriend (e.g., Imagine someone your age who has sex with a boyfriend/girlfriend. How would you describe this person, using the following characteristics: prepared, confident, desperate, etc.?). This measure was adapted from Myklestad and Rise [52], α = .89.
6. *Risk prototype: unprotected sex (14 items)*. Participants will be asked to indicate which of the same 14 adjectives describe a person who has unprotected sex (e.g., Imagine someone your age who has unprotected sex. How would you describe this person, using the following characteristics: prepared, confident, desperate etc.?). This measure was adapted from Myklestad and Rise [52] α = .89.

##### Student satisfaction

In terms of student (consumer) satisfaction, participants will be asked 7 items to indicate on 4-point Likert scales (e.g., 1 = not very much; 2 = a little bit; 3 = a lot; 4 = very much) how satisfactory the program is (e.g., How much did you learn from this program?). Participants will also be asked to open-endedly respond to three questions (e.g., What did you like best about the program?). Participants will be asked to indicate on a 4-point Likert scale (e.g., 1 = not likely at all; 2 = somewhat likely; 3 = likely; 4 = extremely likely) how likely they would be to recommend this program to a friend (e.g., How likely is it that you would recommend this program to a friend?). These measures were adapted from Scull et al. [34].

##### Fidelity of implementation

Process data will be collected to establish dosage. This data will include the start and end times that a user was in the program and which lessons they participated in. Process data do not include specific answers to any program questions.

#### Teacher measures

Teachers will complete a teacher questionnaire after program implementation to assess their satisfaction with the *Media Aware* program, Teacher Dashboard, and Teacher Training. The teacher questionnaire will consist of approximately 110 items and will cover demographics, program satisfaction, teacher training satisfaction, ease of implementation, program component use, and any suggestions for improvement.

### Statistical analyses

#### Preliminary analyses

Psychometric and descriptive analyses will be conducted to study the reliability, validity, and distributions of key variables. The impact of random assignment on producing equivalent groups between conditions will be evaluated using chi-squared analyses with the demographic characteristics. Missing data will be examined at each time point and handled with an appropriate imputation method, if necessary [53].

#### Outcome analyses

The immediate and short-term effectiveness of *Media Aware* for improving student outcomes will be examined using intention-to-treat analyses. Hierarchical linear modeling (with random intercepts at the school/teacher level) will likely be used to account for conditional nonindependence in outcome measures at posttest and follow-up using the SAS MIXED procedure and will examine multiple observations over time. An independent statistician will conduct interim analyses after all posttests are complete, and the PI and Co-Investigator (Co-I) will have access to the interim results. Given the minimal risk of the intervention, it is not anticipated that the trial will be terminated following these interim analyses. However, if the interim analyses suggest the possibility of any adverse effects of the trial on participants, the PI and Co-I will share the findings with the IRB chair and funder to determine how to proceed and whether or not the trial should be terminated. The PI and funder would make the final decision to terminate the trial, if necessary.

The model for the mean of each outcome will likely contain level 1 fixed effects representing the influence of (1) the student’s pretest score and (2) gender and level 2 fixed effects of condition. Student variables found to be nonequivalent between groups will be included as covariates in these models. The effect sizes will be calculated by dividing the appropriate contrast parameter by the sample standard deviation of the outcome. These analyses should reveal if there is a significant improvement in students’ sexual health and media outcomes after having received the program as compared with the delayed-intervention group. Follow-up analyses or structural equation modeling may be warranted. Qualitative analyses (media deconstruction skills) will be coded by research team members who will be blinded to intervention assignment. Unblinding will not occur.

Moderator analyses will examine subpopulations, likely defined by the three categorical variables of gender, relationship status, and prior sexual experience. These findings will provide evidence regarding how the effectiveness of the program varies as a function of student characteristics. Measures of program dosage and interactivity will be examined to see if differing quantity or quality of implementation can explain the findings. It is expected that results from this study will be prepared for publication in scholarly journals and updated in www.ClinicalTrials.gov.

### Ethics

#### Vulnerable population

Students in grades 9 and 10 will be recruited for this study using regulations set forth by the Health and Human Services Regulations regarding the Protection of Human Subjects. The study team will obtain parental permission for the students to participate in the research study, as well as student assent. The parent permission and student assent forms will be available in English and Spanish. Researcher and IRB Chair contact information will be printed on the forms in case parents or students have questions about the study.

#### Data and safety monitoring

To every extent possible, confidentiality will be assured to all participants. Project staff members are required to sign a confidentiality agreement immediately upon beginning employment. All project staff are required to complete training on the protection of human subjects in research. Participants will be given randomly assigned ID numbers to be used on the questionnaires. A separate linking list that includes names and ID numbers will be kept confidentially on a secure server that is only accessible to project members and the Systems Administrators (who are trained in the conduct of ethics research with human participants). After the project has ended and the data have been verified, the linking list will be destroyed, and the data will be rendered anonymous.

Research staff members will scan the incoming online questionnaire data on a regular basis and will be instructed to record any problems seen or concerns about any participants as noted from their responses. The PI will review and respond to these records immediately. Most data from the questionnaires are in the form of Likert scales, which means that participants cannot input anything other than a number response. However, some questions include open-ended fields. It is possible that participants might type identifying information (e.g., names) in the fields. If identifying information has been shared by the participant, it will be remedied immediately.

This is a minimal risk study, and we do not anticipate any study-related adverse events. However, any adverse events that occur will be evaluated and addressed by the PI and study team and reported to the IRB promptly. Serious adverse events (SAEs) will be evaluated within 24 h, and any other anticipated problems or events will be evaluated within 72 h; reporting to the IRB will occur within 2 weeks. If the research staff becomes aware of an SAE, the PI will provide written documentation of the SAE to the IRB Chair. The IRB Chair will provide a report of the SAE and its resolution to both the NIH Program Officer and the PI.

## Discussion

It is important that sexual health education programs be rigorously evaluated to ensure that they are effective in enhancing student health. The program being evaluated in this study, *Media Aware*, has the potential to be an innovative evidence-based program that high schools can implement to promote adolescent sexual and relationship health. In addition, this program has the potential to enhance adolescents’ critical thinking about media messages. The study design, specifically including students’ data collection at three time points (pretest, immediate posttest, and 3 months follow-up), allows for evaluation of the immediate effects of the program on student outcomes, as well as any behavioral effects that may occur during the 3 months after the students receive the program. In addition, this study will gather student and teacher feedback on the program, which will help determine the likelihood of program adoption by schools.

### Potential barriers to study recruitment

There are a few potential barriers to study recruitment. First, it can be difficult to obtain school approval to allow research studies to take place. District or school policy may not allow research studies to be conducted in the schools, and some do not allow the collection of data pertaining to sexual health, specifically. If research is permitted in schools, there may be a rigorous approval process, which includes demonstrating that the research will not cut into instructional time. Schools will be informed that this program can be used as a substitute for other sexual health education lessons and should take less time to complete than traditional sexual health education programs.

Second, some schools require a period during which parents can review a sexual health education curriculum prior to it being taught in the classroom. Parental review of traditional print materials often requires parents to come to the school or district offices to review the curriculum materials, which can be a burden on parents, particularly if they are working and have to visit the school during the workday. Furthermore, there is no way for parents to know exactly how a teacher-led program will be implemented in the classroom (e.g., teachers could add their own content or opinions). The self-contained, web-based nature of *Media Aware* is expected to streamline the parental review process by allowing teachers to send parents a link to the program via an email for review. Furthermore, parents can be assured that the content they review is exactly what their child will be exposed to in class.

Third, schools may also decline to allow this research study to be conducted in their school because changing a school curriculum may elicit teacher pushback. Implementing a new program may require additional effort on the part of their teachers. Teachers have limited class time in which to cover material mandated by the state and school district, and they may view learning a new curriculum as a difficult task. However, given the web-based nature of *Media Aware,* teachers do not have to teach a new curriculum. This feature of utilizing web-based program delivery should minimize teacher resistance to implementing a new curriculum.

### Study strengths and limitations

There are many strengths to the study design. The study is designed as a randomized controlled trial, a rigorous design for program evaluation. Randomization will increase the likelihood that the intervention group and delayed-intervention group are evenly balanced with respect to many characteristics like student diversity and socioeconomic status of the students in the school district. Because only one teacher per school will be participating in the study, there will be little chance of cross contamination between groups. Participating teachers and students from one school will likely have little to no contact with teachers and students in another school. Because the questionnaires will be completed during school hours, study attrition is minimized.

### Implications for the field

This study has many implications for the field of sexual health education. This study will evaluate the feasibility and efficacy of using web-based programming to deliver comprehensive sexual health and media literacy education (MLE) to promote students’ sexual health outcomes. There are few, if any, evidence-based comprehensive sexual education programs for high school students that use an MLE approach, and, to the best of our knowledge, none that used a web-based format to deliver content. Additionally, this program expands the definition of comprehensive sexual health to include topics such as gender, consent, healthy relationships, and communication with partners, parents, and medical providers.

### Trial status

The protocol is Version 1, dated July 29, 2019. The trial is registered on ClinicalTrials.gov, NCT04035694, with public title Media Aware High School Study and scientific title Web-based High School Media Literacy for Healthy Relationships. Secondary identifiers are iRT IRB 19-002-1-EFF and NICHD NIH R44HD088254. The date of first enrollment was September 5, 2019. The trial is currently recruiting; recruitment is slated to be completed by the end of the 2019–2020 school year. Enrollment is in the USA; the problem of study is adolescent sexual health. The trial sponsor is innovation Research & Training (iRT), which may be contacted for public queries (5316 Highgate Drive, Suite 121, Durham, North Carolina, USA 27713).

## Supplementary information

**Additional file 1:** SPIRIT 2013 checklist: recommended items to address in a clinical trial protocol and related documents.

## Data Availability

The datasets generated and/or analyzed during the current study may be available from the corresponding author on reasonable request.

## Abbreviations

DSMP: Data and Safety Monitoring Plan
HIPAA: Health Insurance Portability and Accountability Act
ICC: Intraclass correlation coefficient
ID: Identification
IRB: Internal Review Board
iRT: innovation Research & Training
MLE: Media literacy education
NICHD: Eunice Kennedy Shriver National Institute of Child Health and Human Development
NIH: National Institutes of Health
PI: Principal Investigator
SAE: Serious adverse event
